# A deep learning analysis for dual healthcare system users and risk of opioid use disorder

**DOI:** 10.1038/s41598-024-77602-4

**Published:** 2025-01-29

**Authors:** Ying Yin, Elizabeth Workman, Phillip Ma, Yan Cheng, Yijun Shao, Joseph L. Goulet, Friedhelm Sandbrink, Cynthia Brandt, Christopher Spevak, Jacob T. Kean, William Becker, Alexander Libin, Nawar Shara, Helen M. Sheriff, Jorie Butler, Rajeev M. Agrawal, Joel Kupersmith, Qing Zeng-Trietler

**Affiliations:** 1 Washington DC VA Medical Center, Washington, DC USA; 2 Biomedical Informatics Center, George Washington University, Washington, DC USA; 3 VA Connecticut Healthcare System, West Haven, CT USA; 4 Yale School of Medicine, New Haven, CT USA; 5 Georgetown University School of Medicine, Washington, DC USA; 6 MedStar Health, Washington, DC USA; 7 The University of Utah, Salt Lake City, UT USA

**Keywords:** Deep neural network, Explainable AI, Opioid use disorder, Dual-system use, Interaction, Health care, Risk factors

## Abstract

The opioid crisis has disproportionately affected U.S. veterans, leading the Veterans Health Administration to implement opioid prescribing guidelines. Veterans who receive care from both VA and non-VA providers—known as dual-system users—have an increased risk of Opioid Use Disorder (OUD). The interaction between dual-system use and demographic and clinical factors, however, has not been previously explored. We conducted a retrospective study of 856,299 patient instances from the Washington DC and Baltimore VA Medical Centers (2012–2019), using a deep neural network (DNN) and explainable Artificial Intelligence to examine the impact of dual-system use on OUD and how demographic and clinical factors interact with it. Of the cohort, 146,688(17%) had OUD, determined through Natural Language Processing of clinical notes and ICD-9/10 diagnoses. The DNN model, with a 78% area under the curve, confirmed that dual-system use is a risk factor for OUD, along with prior opioid use or other substance use. Interestingly, a history of other drug use interacted negatively with dual-system use regarding OUD risk. In contrast, older age was associated with a lower risk of OUD but interacted positively with dual-system use. These findings suggest that within the dual-system users, patients with certain risk profiles warrant special attention.

**Supplementary Information:**

The online version contains supplementary material available at 10.1038/s41598-024-77602-4.

## Introduction

Opioids are widely prescribed to millions of Americans with chronic and acute pain. However, the misuse of prescription opioids and the development of opioid use disorder (OUD) have become significant public health crises in the United States over the past two decades. Between 1999 and 2010, there was a sharp increase in opioid prescribing in the U.S., which has led to a dramatic increase in prescription opioid-related overdose deaths^1^. Since 2012, tighter regulation has resulted in a steady decline in opioid prescription in most healthcare settings^2^. OUD, however, did not decline at the same rate^3^. According to the CDC, drug overdose deaths have been increasing from 2019 to 2022, with 107,941 drug overdose deaths reported in 2022^4^.

Similarly, the opioid epidemic has greatly impacted active-military personnel and veterans, reflected by the rising rates of OUD and overdose deaths leading up to 2012^5,6^. The fourth wave of the overdose crisis, driven by synthetic opioids (such as illicitly manufactured fentanyl), is also significantly impacting veterans^6,7^.

In response, the U.S. Veterans Administration (VA) implemented a series of programs to address the issue. For example, with the Opioid Safety Initiative (OSI), an evidence-based guideline on using opioids in the management of chronic pain, the VA has dramatically reduced opioid prescriptions, with only 7.9% of patients receiving these medications in 2021 compared to 22% in 2013^8^. Additional harm reduction programs around OUD have also been implemented in the VA, including syringe service programs (SSPs) and Opioid Overdose Education & Naloxone Distribution (OEND), providing essential education and patient-centered care to veterans, directly working to prevent deadly infections and save lives^9^. Another example is a program called Stepped Care for Opioid Use Disorder Train-the-Trainer (SCOUTT) Initiative to improve access to medications for the treatment of opioid use disorder (MOUD) in primary care, pain management, and mental health clinics.

While significant efforts have been made and progress has been reported in promoting safe opioid use and decreasing opioid-related mortality, a challenge has been the care coordination when patients have access to multiple healthcare systems, or multiple sources of opioid prescriptions. Studies has shown that such fragmented care can leave patients at a higher risk of opioid use and misuse, which may be due to lack of information sharing between healthcare systems^10,11^. This challenge is important for the VA, as many VA enrollees also receive outside care via Veterans Choice Program (VCP)/Veterans Community Care Program (VCCP), which are paid for by the VA. Though dual-system care increased veteran access to community providers, it also added another layer of responsibility for the VA to understand its impact on opioid use. Previous research, including our own studies^12,13^, showed that veterans receiving care from two or more systems faced a heightened risk of OUD. Our research showed that dual-system users differed from mono users in being younger, more likely to be female and minorities, and had more comorbid conditions such as post-traumatic stress disorder (PTSD), depression, chronic pain, or traumatic brain injury (TBI). While baseline conditions played a role, our analysis showed that dual-system users received more opioids and had higher OUD rates even after adjusting with these factors. Thus, the underlying causes of the elevated OUD risk for dual-system users remain unclear, particularly how individual patient factors affect this vulnerability.

Traditionally, differential effects are analyzed using statistical interaction or regression mixture models. The rise of artificial intelligence (AI), especially deep neural network (DNN) models, provided us with a new approach. Literature has shown that when trained on large datasets, DNNs are particularly capable of modeling complex, non-linear relationships without making assumptions of the variable independence or distribution^14^. However, since DNN models often have a large number of parameters, they are difficult to interpret and are thus sometimes called black box models. To tackle this problem, our research team has developed and validated an explainable AI method^15,16^, allowing the assessment of an individual feature’s contribution, as well as the interactions between features that are captured by DNN models.

One challenge we face in analyzing OUD using medical record data is that while it has been widely reported, OUD is often under coded^17^. A number of studies, including one from our team, have developed natural language processing (NLP) methods to identify OUD from clinical notes^18–23^. NLP systems generally consist of either hard-coded rules, trained machine learning models, or both^18^. The development of NLP tools allows us to capture a fuller extent of the OUD problem.

While previous research has demonstrated a heightened risk of opioid use disorder (OUD) among dual-system enrollees, this study delved into how patient demographics and clinic characteristics interact with dual-system use to influence OUD outcomes. We assembled a cohort of veterans from VA Medical Centers in Washington, D.C., and Baltimore, and employed a DNN model to analyze the association between dual-system use and OUD. OUD was determined using NLP from clinical notes and ICD-9/10 diagnoses. By leveraging a novel explainable AI approach, we sought to identify specific subgroups of dual-system enrollees who are particularly vulnerable to OUD. Our findings could inform policymakers in developing targeted interventions to address this pressing public health issue.

## Methods

### Study population

**Fig. 1:**
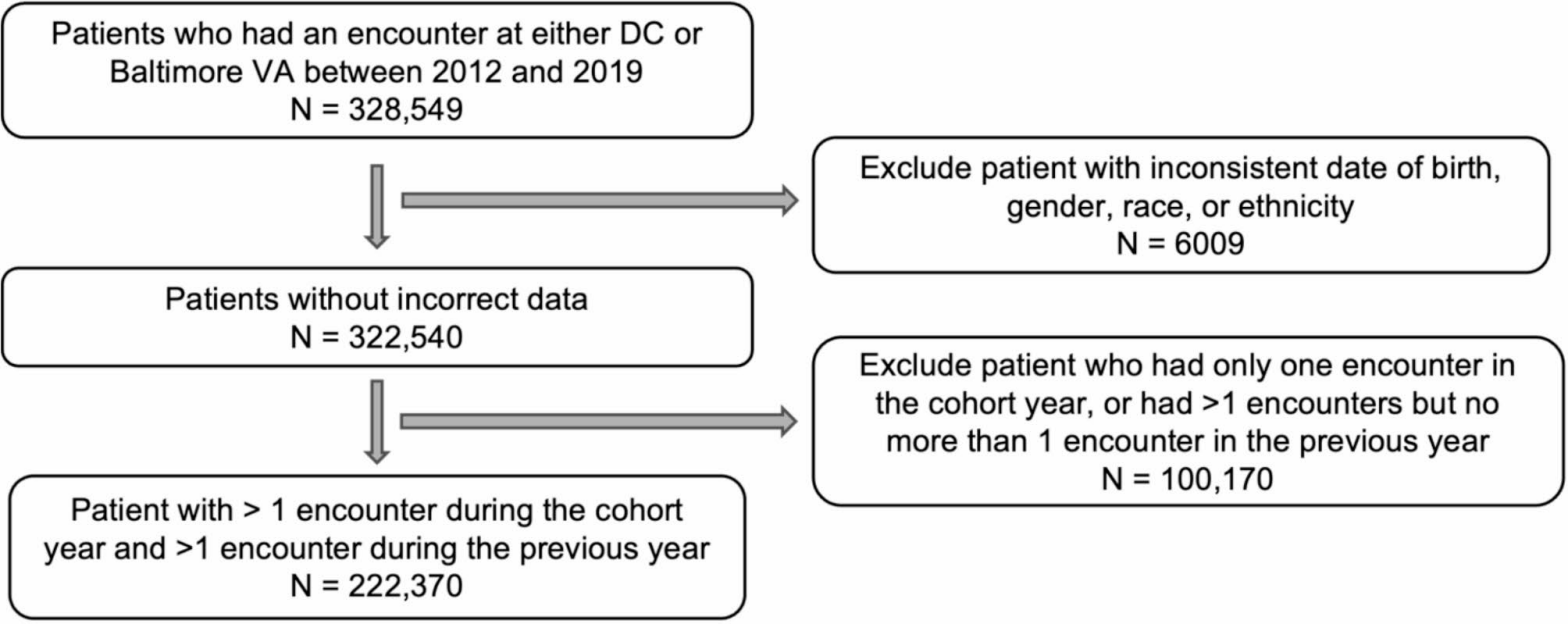
Cohort selection flow chart.

Our study cohort consists of 222,370 distinct patients who received outpatient care between 2012 and 2019 (VA IRBnet protocol #1607134). Active patients who had at least one encounter of any type in the VA (in DC or Baltimore) within a calendar year (2012–2019) were enrolled, and patients with no encounter in the previous calendar year were excluded. Additionally, patients with multiple or inconsistent dates of birth, gender, race, or ethnicity were excluded due to data quality concerns (Fig. 1). Given dual-system user status changes over time, each year is treated as a separate cohort. This resulted in a total of 856,299 patient instances.

All data were derived from electronic health records (EHR) from the VA Corporate Data Warehouse (CDW). Research was performed within the secure VA Informatics and Computing Infrastructure (VINCI) platform^24^.

## Predictors

We defined dual healthcare system users as those with VCP/VCCP encounters within the calendar year by use of a VA stop code designated for the community care program, or VCP/VCCP note. Patients without any VCP/VCCP encounter in a calendar year were defined as “mono” users.

For each year, we used January 1st of the cohort year as the index date to extract additional demographic (e.g., age, gender, race, etc.) and clinical information. Age is calculated at the index date. Baseline comorbidities were defined using diagnostic codes (Supplementary Table 1) documented any time before the index date. Other drug disorders include cannabis-related disorders, nicotine dependence, cocaine-related disorders and other stimulant related disorders. Prior opioid prescription is defined as receipt of any opioid prescriptions prior to the index date. Opioid medications included the following: codeine, fentanyl, hydrocodone, hydromorphone, levorphanol, meperidine, morphine, oxycodone, oxymorphone, pentazocine, tapentadol and tramadol.

## Study outcomes

The study outcome was OUD during the calendar year (within 12 months of the index date). OUD was defined in two ways: (1) using the ICD 9/10 codes of 304.0, 304.7, 305.5, and F11; and (2) the OUD-related issues documented in clinical notes that are extracted by the NLP tool described below.

## NLP application

To identify patients with problematic opioid use-related concerns documented in their clinical notes, we developed an NLP classifier that uses both rule-based and machine learning methods^23^. After reviewing sample notes, two project members identified 36 key phrases relevant to problematic opioid use concern documentation. Using snippets (a span of clinical note text containing one of the key phrases), the team developed a support vector machine (SVM) model to identify notes documenting problematic opioid use concerns. Also using snippets, the team built a library of regular expressions matching relevant template data (e.g., “[x] substance abuse and/or dependence”) and relevant standard clinical text (e.g., “current opioid dependence”). Leveraging both the SVM and regular expressions, the NLP classifier achieved 96.6% specificity, 90.4% precision/PPV, 88.4% sensitivity/recall, and 94.4% accuracy on an unseen (i.e. not used in classifier development) snippet dataset.

## DNN modeling

**Fig. 2:**
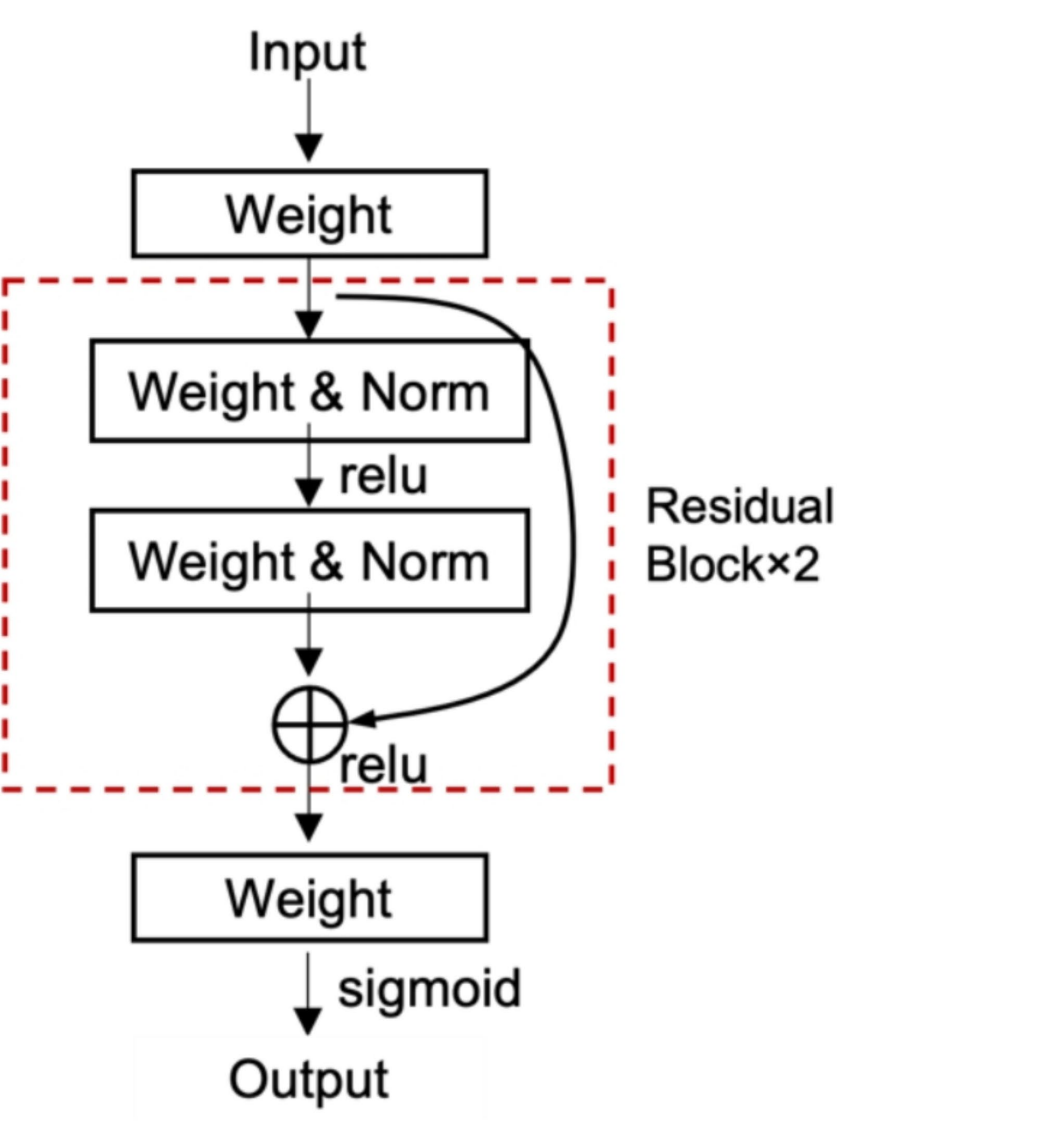
DNN Architect.

The DNN model is assembled with a variation of the residual network (ResNet) model^25^, as shown in Fig. 2. The input variables undergo a linear transformation, followed by two residual blocks. Each residual block consists of two feed-forward layers with layer normalization and ReLU activation, and a skip connection to add the residual from the previous layer. The advantage of the ResNet model is that it is more stable and efficient for training, as the incorporation of skip connections effectively prevents the vanishing gradient problem. The final layer is activated with the sigmoid function σ, so that the output value is always between 0 and 1.

The dataset was split into training, validation, and testing sets in a ratio of 64%, 16%, and 20%, respectively. Due to the rarity of the observed outcome, an imbalance between positive and negative instances existed in the dataset. To address this issue, the Random Under-Sampling (RUS) algorithm was used to randomly select data points from the non-OUD class matching the size of the OUD class during training. The validation set and test set remained unchanged representing the original population.

We employed a binary cross-entropy loss function to guide our model’s training. The F1 score, which balances precision and recall, served as our primary performance metric. Stochastic gradient descent was used to optimize the model’s parameters. To prevent overfitting, we implemented a dropout rate of 0.25 and an early stopping mechanism that halted training when the model’s validation F1 score ceased to improve for ten epochs. To assess the model’s uncertainty, we calculated the confidence interval of the area under the curve (AUC) based on the number of positive and negative samples^26^.

### Statistical analyses

We summarized the baseline characteristics of patients with and without OUD diagnoses. To evaluate the balance between these groups, we computed absolute standardized differences (ASDs) for each comparison, with an ASD greater than 10% indicating significant imbalance. For outcome prediction, we performed multivariate logistic regression analysis using all relevant predictors as a benchmark against the DNN model. Statistical analyses were conducted using Python, employing the scikit-learn, SciPy, and Matplotlib libraries for data analysis and visualization.

## Impact and impact scores

Because DNN models are often considered black boxes, our team developed a scoring method to measure the impact of features on model output^15,27^. The output score, defined as the impact score, is similar to the coefficient of a logistic regression model, which measures the association between changes in the predictor value and changes in the output. For binary variables, the score measures the difference between the output when the feature is absent and the output when the feature is present. For continuous variables, the score measures the impact of a one-unit increase in the variable value.

## Interaction and interaction score

Similarly, we designed the interaction score to measure the interaction between two variables^28^. Conceptually, we define the interaction between two variables as the residual impact of changes in both variables simultaneously subtracting the impact of each individual variable.

### Confidence interval and bootstrapping

To estimate the confidence intervals of the impact scores and interaction scores of our model, we employed the nonparametric percentile bootstrap method. In this approach, we resampled the original data with replacement 200 times, trained the model, and independently calculated the impact scores and interaction scores. The 95% confidence interval was determined as the range between the 2.5th and 97.5th percentiles of the calculated scores for each feature.

## Results

Among the 856,299 patient instances analyzed from 2012 to 2019, 16,582 (2%) were identified as having an ICD diagnosis of OUD, while 144,695 (16.9%) were classified as having OUD by NLP. Notably, 144,695 (17.1%) patients met both criteria, indicating a significant underdiagnosis of OUD, as our NLP approach identified over eight times the number of cases compared to ICD-based diagnosis.

A comparison of demographic and clinical characteristics between patients with and without OUD is presented in Table 1. In summary, patients with OUD tend to be younger, were more likely to be Black and single, and were more likely to have dual-system use, a history of opioid prescription, alcohol use disorder, tobacco use disorder and other substance use disorders (including cannabis-related disorders, nicotine dependence, cocaine-related disorders and other stimulant related disorders).

**Fig. 3:**
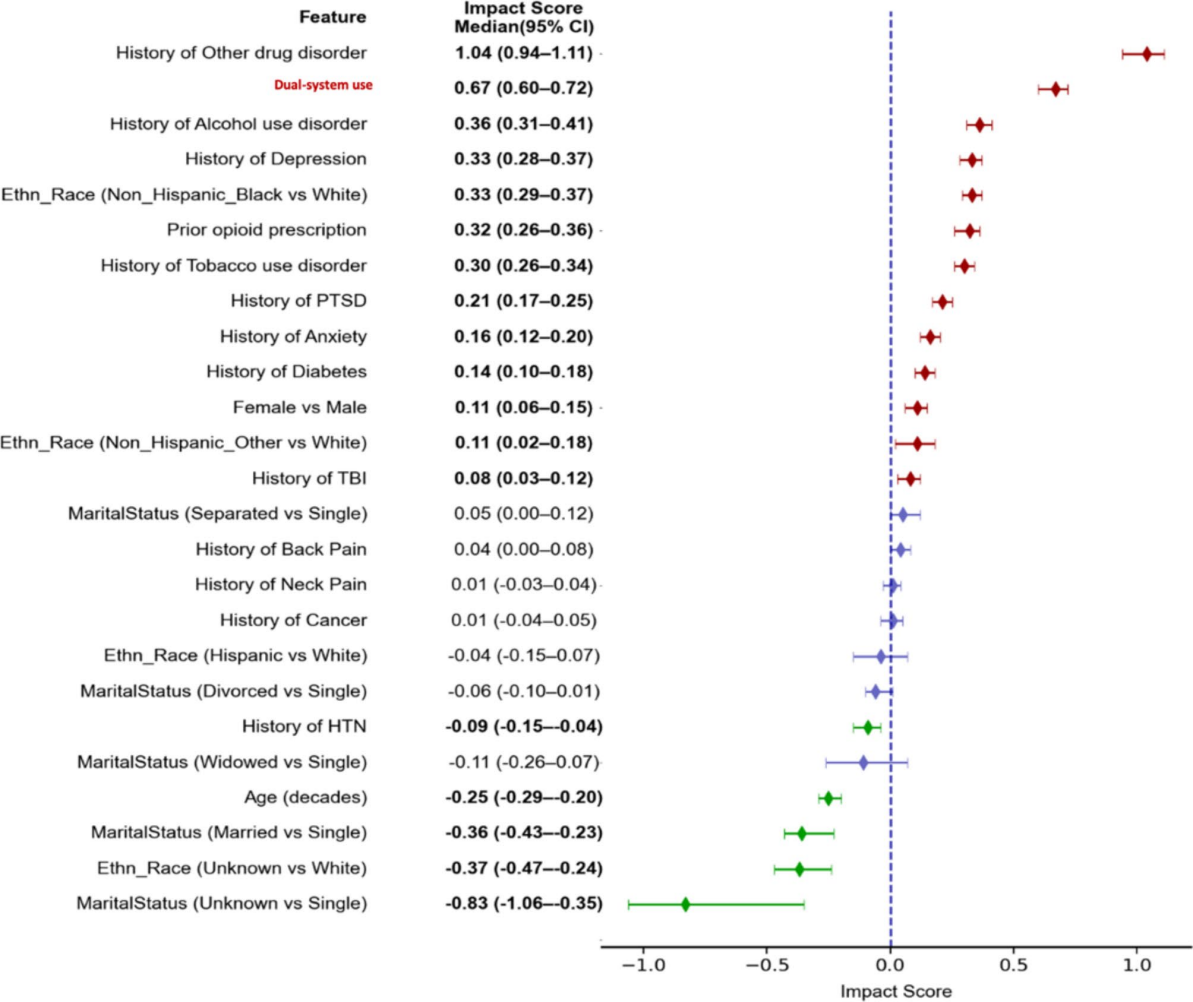
Impact Scores and 95% Confidence Intervals of Predictors on OUD Outcomes. HTN = Hypertension; OUD = Opioid Use Disorder; PTSD = Post Traumatic Stress Disorder; TBI = Traumatic Brain Injury.

Using these predictors for OUD outcomes in our DNN model, we achieved a 78.2% (CI: 77.9-78.5%) AUC for testing (Table 2), which represents a marginal improvement from the logistic regression model with an AUC of 77%. We calculated the impact score for each feature, where positive values indicate increased OUD risk. As illustrated in Fig. 3, a history of other substance use disorders is the major risk factor for OUD, and age is associated with a lower risk of OUD, which is consistent with previous reports^5,6,29^. Dual system use is also on the top list of risk factors associated with elevated risk of OUD. These findings are consistent with the logistic regression results, which demonstrated an odds ratio of 2.09 (95% CI: 1.96–2.22) for dual-system use after adjusting for all other covariates. Furthermore, the impact scores demonstrated a strong correlation with the logistic regression coefficients, with a correlation coefficient of 0.99.

**Fig. 4:**
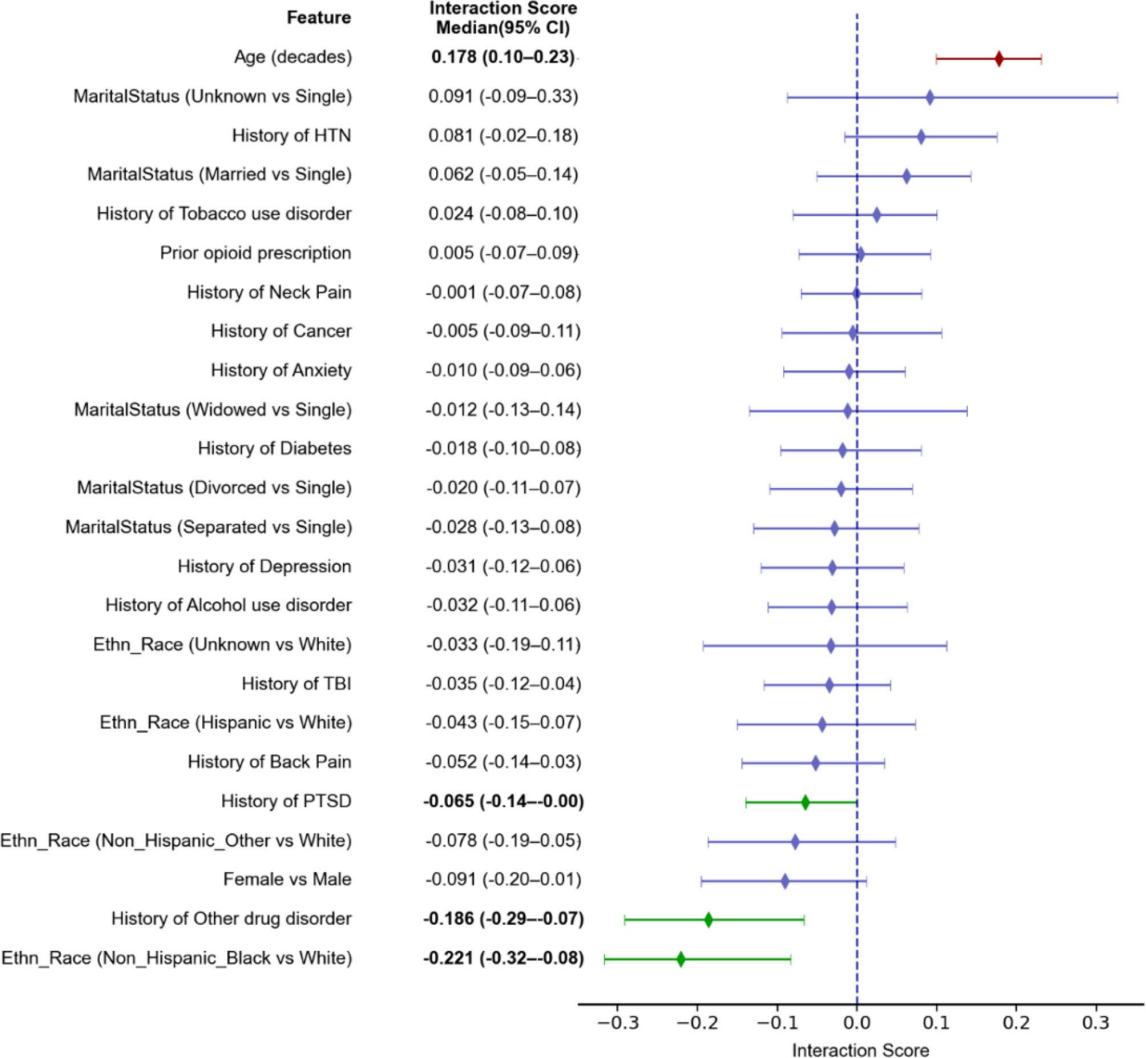
Interaction Scores and 95% Confidence Intervals for Feature Interactions with Dual-System User on OUD Outcomes. HTN = Hypertension; OUD = Opioid Use Disorder; PTSD = Post Traumatic Stress Disorder; TBI = Traumatic Brain Injury.

The DNN analysis provided additional insights by calculating interaction scores, which analyze the feature interactions between other predictors and dual-system use status at both population and individual levels. The average interaction score (Fig. 4) revealed a significant positive interaction between age and dual-system use. This suggests that while older age is generally associated with a decreased risk of OUD, individuals who are older and enrolled in dual-system programs may be at a heightened risk. In contrast, races such as Non-Hispanic Black or Non-Hispanic Other, as well as a history of other drug disorders and prior PTSD diagnosis, were found to interact negatively with dual-system use status.

**Table 1:** Demographics of groups with and without OUD based on NLP or ICD codes.

	Opioid use disorder		ASD (%)
	No	Yes	
	*N* = 709,611	*N* = 146,688	
Age	61.9 ± 17.0	56.8 ± 14.2	**32**
Dual-system user	54,489 (8%)	22,982 (16%)	**25**
Female	99,325 (14%)	22,916 (16%)	5
Race			
Non-hispanic white	324,987 (46%)	46,937 (32%)	**29**
Hispanic	16,700 (2%)	3146 (2%)	1
Non-hispanic black	278,841 (39%)	87,740 (60%)	**42**
Non-hispanic other	30,933 (4%)	5371 (4%)	4
Unknown	58,150 (8%)	3494 (2%)	**26**
Marital status			
Single	117,994 (17%)	35,993 (25%)	**20**
Divorced	142,313 (20%)	41,879 (29%)	**20**
Married	347,810 (49%)	48,335 (33%)	**33**
Separated	29,482 (4%)	12,010 (8%)	**17**
Unknown	22,690 (3%)	910 (1%)	**19**
Widowed	49,322 (7%)	7561 (5%)	8
Prior opioid prescription	336,428 (47%)	99,550 (68%)	**42**
Comorbidity			
Alcohol use disorder	95,641 (13%)	62,532 (43%)	**69**
Anxiety	150,629 (21%)	58,060 (40%)	**41**
Back pain	318,722 (45%)	87,035 (59%)	**29**
Cancer	122,294 (17%)	19,947 (14%)	**10**
Diabetes	212,854 (30%)	86,098 (59%)	3
Depression	203,234 (29%)	43,941 (30%)	**60**
Hypertension	435,223 (61%)	92,422 (63%)	3
Neck pain	200,018 (28%)	61,288 (42%)	**29**
Other drug disorder*	59,672 (8%)	59,825 (41%)	**81**
PTSD	121,873 (17%)	52,920 (36%)	**44**
TBI	38,192 (5%)	15,269 (10%)	**19**
Tobacco use disorder	159,410 (22%)	69,949 (48%)	**55**

PTSD, Post-Traumatic Stress Disorder; TBI, Traumatic Brain Injury.

* Including cannabis-related disorders, nicotine dependence, cocaine-related disorders and other stimulant related disorders.

ASD values greater than 10% are in bold, indicating significant differences between the groups.

**Table 2:** DNN performances on training, validation and test datasets.

	F1	Accuracy	Precision	Recall	AUC
Training	0.699	0.708	0.72	0.679	0.783
Validation	0.461	0.727	0.349	0.681	0.783
Test	0.462	0.728	0.35	0.68	0.784

## Discussion

The explainable AI analyses confirmed that dual-system use is associated with OUD in our cohort. This finding aligns with previous research identifying dual-system use as a risk factor^13^. Known risk factors such as prior drug abuse and prior opioid prescription have also been validated^30,31^.

The interaction analysis of OUD with other covariates revealed that the combined impact of dual-system use with a number of conditions (e.g., PTSD, abuse of other drugs) is less than their additive impacts. Conversely, older patients who are dual system users are at a higher risk for OUD.

Interestingly, predictors with a negative interaction score regarding dual user status tend to be those associated with a positive impact score. Conversely, predictors with a positive interaction score concerning dual-system use are often associated with a negative impact score. This suggests that dual-system use disproportionately affects individuals not typically seen as high-risk. In particular, patients with lower OUD risk profiles, such as older individuals or those with no substance use history, may be more susceptible to developing OUD when enrolled in dual systems compared to those receiving care solely within the VA. This may be attributed to a lack of targeted OUD prevention or treatment efforts for lower-risk patients and the increased likelihood of opioid prescriptions for chronic pain management in uncoordinated care settings.

For our cohort assembly, we did not exclude patients with existing OUD. In general, patients with a prior OUD diagnosis will be more likely to receive OUD diagnoses in subsequent years^32,33^. In our cohort, 35% of the cases were incident OUD, while 65% were prevalent OUD. Our research aimed to determine whether dual-system healthcare utilization is linked to an increased risk of OUD, for all patients with or without prior OUD history, and to identify which populations were particularly at risk. Therefore, we did not limit the outcome to incident OUD diagnosis. However, we conducted a sensitivity analysis excluding patients with a prior OUD history, and the results remained consistent. In this adjusted analysis, dual-system use emerged as the top risk factor, with an impact score of 0.68, slightly higher than that of other substance use disorders (impact score = 0.67). Age also showed a strong interaction with dual-system use, indicating that older patients with no history of OUD might be more vulnerable to developing OUD when enrolled in dual-system care.

One limitation of this study is the dual use status was limited to VA-paid community care, while Veterans may seek care outside of the VA independently. Another limitation is the imperfect nature of OUD diagnosis in EHR. The stigma surrounding OUD may discourage individuals from seeking care^34^. Though we tried to identify underdiagnosed OUD by NLP, this does not equate to an OUD diagnosis. Nevertheless, our NLP classifier achieved over 94.4% accuracy, as confirmed by medical expert annotation. Within our cohort, 16,852 patient instances had an ICD-based OUD diagnosis, of which 88% were corroborated by NLP. At the same time, the NLP identified almost eight times more patients with possible OUD conditions. Given the reality of under-coding of OUD in EHRs^17^, the NLP program provides additional insights into OUD prevalence among Veterans. Our analysis confirmed that dual-system users are more likely to have OUD identified by both ICD and NLP.

A future direction we plan to explore is analyzing the underlying causes of interactions that increase OUD risks. In addition, social and community factors play important roles in the prevention of OUD. We hope to include those factors in future analyses. While prior opioid prescriptions are known to be associated with a higher risk of OUD, we aim to further investigate how treatment dose (measured in morphine milligram equivalents) and treatment duration affect both incident and prevalent OUD, particularly among patients enrolled in dual systems.

## Electronic supplementary material

Below is the link to the electronic supplementary material.

Supplementary Material 1

## Data Availability

The data that support the findings of this study are derived from the Veterans Health Administration’s Clinical Data Warehouse. Restrictions apply to the availability of these data, which were used under license for this study. Data are available to VA researchers from the corresponding author on reasonable request.
