# Supplementary material for: A deep learning analysis for dual healthcare system users and risk of opioid use disorder
Source: Sci Rep. 2025 Jan 29;15:3648. doi: 10.1038/s41598-024-77602-4 (PMC11779826; doi:10.1038/s41598-024-77602-4)
Supplement: Supplementary file 1 — Supplementary Material 1 [file 41598_2024_77602_MOESM1_ESM.pdf]

A Deep Learning analysis for Dual Healthcare System Users and Risk of Opioid Use Disorder

Ying Yin, PhD[1,2], T Elizabeth Workman, PhD[1,2], Phillip Ma, MD[1,2], Yan Cheng, PhD[1,2], Yijun Shao, PhD[1,2],Joseph L. Goulet, PhD[3,4], Friedhelm Sandbrink[1], Cynthia Brandt[3,4], Christopher Spevak[5] , Jacob T. Kean[7], William Becker[3,4], Alexander Libin[5,6], Nawar Shara[5,6], Helen M Sheriff[1], Jorie Butler[7], Rajeev M Agrawal[6], Joel Kupersmith[5,*], Qing Zeng-Trietler[1,2*]

[1]Washington DC VA Medical Center, Washington, DC, United States,

[2]Biomedical Informatics Center, George Washington University, Washington, DC, United States,

[3] VA Connecticut Healthcare System, West Haven, CT, United States,

[4]Yale School of Medicine, New Haven, CT, United States,

[5]Georgetown University School of Medicine, Washington, DC, United States,

[6]MedStar Health, Washington, DC, United States,

[7]The University of Utah, Salt Lake City, UT;

[*]Qing Zeng-Treitler (zengq@gwu.edu) and Joel Kupersmith(jk1688@georgetown.edu) are co-senior authors and contributed equally

Supplementary Table 1: Diagnosis Codes

Condition	ICD-9CM	ICD-10CM
Alcohol use disorder	305.0%, 303%	F10%
Anxiety	300.0%, 309.24, 309.28	F41%, F43.22, F43.23
Back Pain	720.1, 720.2, 721.2, 721.3, 721.41, 721.42, 722.10, 722.11, 722.31, 722.32, 722.51, 722.52, 722.72, 722.73, 722.82, 722.83, 722.92, 722.93, 724.00, 724.01, 724.02, 724.09, 724.1, 724.2, 724.3, 724.4, 724.5, 724.6, 724.70, 724.71, 724.79, 724.8, 724.9, 737.10, 737.11, 737.20, 737.21, 737.22, 737.29, 737.34, 737.41, 737.42, 738.4, 739.2, 739.3, 739.4, 756.11, 805.3, 805.4, 805.5, 805.6, 805.7, 806.20, 806.21, 806.22, 806.23, 806.24, 806.25, 806.26, 806.27, 806.28, 806.29, 806.30, 806.31, 806.32, 806.33, 806.34, 806.35, 806.36, 806.37, 806.38, 806.39, 806.4, 806.5, 806.60, 806.61, 806.62, 806.69, 806.70, 806.71, 806.72, 806.79, 839.20, 839.21, 839.30, 839.31, 839.40, 839.41, 839.42, 839.49, 839.50, 839.51, 839.52, 839.59, 839.61, 839.69, 839.71, 839.79, 846.0, 846.1, 846.2, 846.3, 846.8, 846.9, 847.1, 847.2, 847.3, 847.4	M40.00, M40.03, M40.04, M40.05, M40.10, M40.12, M40.13, M40.14, M40.15, M40.202, M40.203, M40.204, M40.205, M40.40, M40.45, M40.46, M40.47, M40.50, M40.55, M40.56, M40.57, M41.30, M41.34, M41.35, M43.04, M43.05, M43.06, M43.07, M43.08, M43.14, M43.15, M43.16, M43.17, M43.18, M43.24, M43.25, M43.26, M43.27, M43.28, M43.8X4, M43.8X5, M43.8X6, M43.8X7, M43.8X8, M43.9, M46.04, M46.05, M46.06, M46.07, M46.08, M46.1, M46.44, M46.45, M46.46, M46.47, M46.48, M47.014, M47.015, M47.016, M47.13, M47.14, M47.15, M47.16, M47.24, M47.25, M47.26, M47.27, M47.28, M47.813, M47.814, M47.815, M47.816, M47.817, M47.818, M47.893, M47.894, M47.895, M47.896, M47.897, M47.898, M48.00, M48.04, M48.05, M48.061, M48.07, M48.08, M51.04, M51.05, M51.06, M51.14, M51.15, M51.16, M51.17, M51.24, M51.25, M51.26, M51.27, M51.34, M51.35, M51.36, M51.37, M51.44, M51.45, M51.46, M51.47, M51.84, M51.85, M51.86, M51.87, M53.2X%, M53.2X8, M53.3, M53.84, M53.85, M53.86, M53.87, M53.88, M54.03, M54.04, M54.05, M54.06, M54.07, M54.08, M54.09, M54.14, M54.15, M54.16, M54.17, M54.18, M54.30, M54.31, M54.32, M54.40, M54.41, M54.42, M54.5, M54.6, M54.89, M54.9, M62.830, M96.1, M96.2, M96.4, M99.02, M99.03, M99.04, M99.12, M99.13, M99.14,

		M99.15, M99.16, M99.18, M99.22, M99.23, M99.24, M99.25, M99.26, M99.27, M99.28, M99.29, M99.33, M99.34, M99.35, M99.36, M99.37, M99.38, M99.39, M99.42, M99.43, M99.44, M99.45, M99.46, M99.47, M99.48, M99.49, M99.52, M99.53, M99.54, M99.55, M99.56, M99.57, M99.58, M99.59, M99.62, M99.63, M99.64, M99.65, M99.66, M99.67, M99.68, M99.69, M99.72, M99.73, M99.74, M99.75, M99.76, M99.77, M99.78, M99.79, Q76.2, S03.1XXA, S21.109A, S22.0%, S23.1%, S23.2%, S23.3%, S23.8%, S24.10%, S24.11%, S24.13%, S24.15%, S31.000A, S32.0%, S32.1%, S32.2X%, S33.0%, S33.1%, S33.2%, S33.2XXA, S33.3%, S33.5%, S33.6%, S33.8%, S33.9%, S34.1%, S34.3XXA, S39.01%, S39.92%
Cancer	140-149%, 159%, 164%, 172-176%, 189-196%, 199-208%	Z85%, D03%, D45%, C26%, C37%, C43%, C46%, C50-58%, C60-77%, C80-86%, C88%, C90-96%
Diabetes	250%	E10, E11, E13
Depression	296.2%, 296.3%, 311%	F32%, F33%
Hypertension	401, 401.1, 401.9	I10%
Neck Pain	721.0, 721.1, 722.0, 722.4, 722.71, 722.81, 722.91, 723.0, 723.1, 723.2, 723.3, 723.4, 723.5, 723.6, 723.7, 723.8, 723.9, 738.2, 739.1, 756.2, 784.0, 805.00, 805.01, 805.02, 805.03, 805.04, 805.05, 805.06, 805.07, 805.08, 805.10, 805.11, 805.12, 805.13, 805.14, 805.15, 805.16, 805.17, 805.18, 806.00, 806.01, 806.02, 806.03, 806.04, 806.05, 806.06, 806.07, 806.08, 806.09, 806.10, 806.11, 806.12, 806.13, 806.14, 806.15, 806.16, 806.17, 806.18, 806.19, 839.00, 839.01, 839.02, 839.03, 839.04, 839.05, 839.06, 839.07, 839.08, 839.10, 839.11, 839.12, 839.13, 839.14, 839.15, 839.16, 839.17, 839.18, 847.0	G44.1, M43.01, M43.02, M43.03, M43.11, M43.12, M43.13, M43.21, M43.22, M43.23, M43.6, M43.8X1, M43.8X2, M43.8X3, M46.01, M46.02, M46.03, M46.41, M46.42, M46.43, M47.011, M47.012, M47.013, M47.021, M47.022, M47.11, M47.12, M47.22, M47.23, M47.811, M47.812, M47.891, M47.892, M48.01, M48.02, M48.03, M50.00, M50.01, M50.02%, M50.03, M50.10, M50.11, M50.12%, M50.13, M50.2%, M50.30, M50.31, M50.32%, M50.33, M50.80, M50.81, M50.82%, M50.83, M50.90, M50.91, M50.92%, M50.93, M53.0, M53.1, M53.81, M53.82,

		M53.83, M54.00, M54.01, M54.02, M54.11, M54.12, M54.13, M54.2, M54.81, M67.88, M95.3, M99.01, M99.10, M99.11, M99.20, M99.21, M99.30, M99.31, M99.40, M99.41, M99.50, M99.51, M99.60, M99.61, M99.70, M99.71, M99.81, Q76.5, S12.0%, S12.1%, S12.2%, S12.3%, S12.4%, S12.40%, S12.5%, S12.50%, S12.6%, S12.60%, S12.9X%, S13.0XXA, S13.1%, S13.2%, S13.4%, S13.8%, S13.9%, S14.101%, S14.102%, S14.103%, S14.104%, S14.105%, S14.106%, S14.107%, S14.111%, S14.112%, S14.113%, S14.114%, S14.115%, S14.116%, S14.117%, S14.121%, S14.122%, S14.123%, S14.124%, S14.125%, S14.126%, S14.127%, S14.131%, S14.132%, S14.133%, S14.134%, S14.135%, S14.136%, S14.137%, S14.151%, S14.152%, S14.153%, S14.154%, S14.155%, S14.156%, S14.157%, S14.40%, S14.50%, S14.60%, S16.1%
Other drug disorder	304.1-304.6,304.8-304.9, 305.2-305.4, 305.6-305.9	F12-F19
PTSD	309.81	F43.1%
TBI	800.04, 800.10, 800.21, 800.35, 800.40, 800.49, 800.50, 800.60, 800.63, 800.65, 800.73, 800.75, 800.79, 800.81, 800.84, 800.91, 800.94, 800.96, 801.01, 801.14, 801.24, 801.32, 801.41, 801.44, 801.46, 801.51, 801.56, 801.61, 801.63, 801.70, 801.73, 801.75, 801.81, 801.93, 803.06, 803.14, 803.16, 803.20, 803.22, 803.31, 803.41, 803.42, 803.52, 803.54, 803.60, 803.65, 803.71, 803.73, 803.82, 803.84, 803.86, 803.89, 803.93, 803.95, 803.99, 804.04, 804.06, 804.14, 804.16, 804.26, 804.31, 804.34, 804.41, 804.54, 804.56, 804.64, 804.66, 804.69, 804.72, 804.74, 804.82, 804.84,	S06.332A, S06.332D, S06.332S, S06.333A, S06.333D, S06.333S, S06.334A, S06.334D, S06.334S, S06.335A, S06.335D, S06.335S, S06.336A, S06.336D, S06.336S, S06.337A, S06.337D, S06.337S, S06.338A, S06.338D, S06.338S, S06.339A, S06.339D, S06.339S, S06.340A, S06.340D, S06.340S, S06.341A, S06.341D, S06.341S, S06.342A, S06.342D, S06.342S, S06.343A, S06.343D, S06.343S,

	804.89, 804.94, 804.96, 850.1, 850.3, 851.00, 851.09, 851.10, 851.13, 851.19, 851.20, 851.22, 851.31, 851.42, 851.49, 851.52, 851.54, 851.59, 851.61, 851.63, 851.72, 851.74, 851.79, 851.80, 851.89, 851.92, 851.95, 852.05, 852.11, 852.16, 852.21, 852.36, 852.46, 852.55, 853.03, 853.05, 853.12, 853.14, 853.19, 854.06, 854.11, 800.01, 800.02, 800.03, 800.15, 800.16, 800.22, 800.23, 800.34, 800.42, 800.43, 800.44, 800.45, 800.61, 800.62, 800.64, 800.89, 800.90, 800.92, 800.95, 800.99, 801.00, 801.02, 801.03, 801.04, 801.05, 801.06, 801.20, 801.21, 801.36, 801.39, 801.40, 801.42, 801.43, 801.45, 801.65, 801.66, 801.71, 801.72, 801.74, 801.85, 801.86, 801.90, 801.99, 803.01, 803.02, 803.03, 803.04, 803.19, 803.21, 803.43, 803.44, 803.45, 803.53, 803.56, 803.59, 803.66, 803.70, 803.80, 803.81, 803.90, 803.91, 803.92, 804.09, 804.10, 804.11, 804.12, 804.29, 804.30, 804.33, 804.35, 804.36, 804.50, 804.51, 804.52, 804.53, 804.65, 804.70, 804.71, 804.86, 804.91, 804.92, 804.93, 850.11, 850.12, 850.4, 850.5, 851.14, 851.15, 851.23, 851.24, 851.25, 851.55, 851.56, 851.64, 851.65, 851.66, 851.75, 851.76, 851.90, 851.91, 851.93, 851.96, 852.00, 852.01, 852.02, 852.03, 852.20, 852.22, 852.23, 852.31, 852.32, 852.33, 852.34, 852.49, 852.59, 853.09, 853.15, 853.16, 854.09, 854.13, 854.14, 854.15, 854.16, 310.2, 800.06, 800.09, 800.19, 800.20, 800.26, 800.30, 800.36, 800.39, 800.41, 800.46, 800.51, 800.53, 800.56, 800.59, 800.66, 800.69, 800.74, 800.76, 800.82, 800.83, 800.93, 801.09, 801.13, 801.19, 801.23, 801.25, 801.26, 801.30, 801.33, 801.35, 801.50, 801.52, 801.59, 801.60, 801.62, 801.64, 801.69, 801.76, 801.82, 801.84, 801.89, 801.92, 801.94, 801.95, 803.00, 803.05, 803.13, 803.15, 803.23, 803.30, 803.32, 803.39, 803.40, 803.49, 803.55, 803.62, 803.69, 803.72, 803.74,	S06.344A, S06.344D, S06.344S, S06.345A, S06.345D, S06.345S, S06.346A, S06.346D, S06.346S, S06.347A, S06.347D, S06.347S, S06.348A, S06.348D, S06.348S, S06.349A, S06.349D, S06.349S, S06.350A, S06.350D, S06.350S, S06.351A, S06.351D, S06.351S, S06.352A, S06.352D, S06.352S, S06.353A, S06.353D, S06.353S, S06.354A, S06.354D, S06.354S, S06.355A, S06.355D, S06.355S, S06.356A, S06.356D, S06.356S, S06.357A, S06.357D, S06.357S, S06.358A, S06.358D, S06.358S, S06.359A, S06.359D, S06.359S, S06.360A, S06.360D, S06.360S, S06.361A, S06.361D, S06.361S, S06.362A, S06.362D, S06.362S, S06.363A, S06.363D, S06.363S, S06.364A, S06.364D, S06.364S, S06.365A, S06.365D, S06.365S, S06.366A, S06.366D, S06.366S, S06.367A, S06.367D, S06.367S, S06.368A, S06.368D, S06.368S, S06.369A, S06.369D, S06.369S, S06.370A, S06.370D, S06.370S, S06.371A, S06.371D, S06.371S, S06.372A, S06.372D, S06.372S, S06.373A, S06.373D, S06.373S, S06.374A, S06.374D, S06.374S, S06.375A, S06.375D, S06.375S, S06.376A, S06.376D, S06.376S, S06.377A, S06.377D, S06.377S, S06.378A, S06.378D, S06.378S, S06.379A, S06.379D, S06.379S, S06.380A, S06.380D, S06.380S, S06.381A,

	803.83, 803.85, 803.94, 803.96, 804.05, 804.13, 804.15, 804.20, 804.23, 804.25, 804.32, 804.39, 804.40, 804.42, 804.49, 804.55, 804.61, 804.73, 804.75, 804.83, 804.85, 804.90, 804.95, 850.2, 850.9, 851.01, 851.06, 851.11, 851.12, 851.21, 851.29, 851.30, 851.32, 851.39, 851.41, 851.51, 851.53, 851.60, 851.62, 851.69, 851.71, 851.73, 851.81, 851.83, 851.86, 851.94, 852.04, 852.10, 852.15, 852.26, 852.29, 852.30, 852.35, 852.40, 852.45, 852.50, 852.54, 852.56, 853.04, 853.06, 853.11, 853.13, 854.03, 854.05, 854.10, 854.12, 854.19, 905.0, 950.1, 959.01, V15.52, 800.00, 800.05, 800.11, 800.12, 800.13, 800.14, 800.24, 800.25, 800.29, 800.31, 800.32, 800.33, 800.52, 800.54, 800.55, 800.70, 800.71, 800.72, 800.80, 800.85, 800.86, 801.10, 801.11, 801.12, 801.15, 801.16, 801.22, 801.29, 801.31, 801.34, 801.49, 801.53, 801.54, 801.55, 801.79, 801.80, 801.83, 801.91, 801.96, 803.09, 803.10, 803.11, 803.12, 803.24, 803.25, 803.26, 803.29, 803.33, 803.34, 803.35, 803.36, 803.46, 803.50, 803.51, 803.61, 803.63, 803.64, 803.75, 803.76, 803.79, 804.00, 804.01, 804.02, 804.03, 804.19, 804.21, 804.22, 804.24, 804.43, 804.44, 804.45, 804.46, 804.59, 804.60, 804.62, 804.63, 804.76, 804.79, 804.80, 804.81, 804.99, 850.0, 851.02, 851.03, 851.04, 851.05, 851.16, 851.26, 851.33, 851.34, 851.35, 851.36, 851.40, 851.43, 851.44, 851.45, 851.46, 851.50, 851.70, 851.82, 851.84, 851.85, 851.99, 852.06, 852.09, 852.12, 852.13, 852.14, 852.19, 852.24, 852.25, 852.39, 852.41, 852.42, 852.43, 852.44, 852.51, 852.52, 852.53, 853.00, 853.01, 853.02, 853.10, 854.00, 854.01, 854.02, 854.04, 907.0, 950.2, 950.3, 959.9	S06.381D, S06.381S, S06.382A, S06.382D, S06.382S, S06.383A, S06.383D, S06.383S, S06.384A, S06.384D, S06.384S, S06.385A, S06.385D, S06.385S, S06.386A, S06.386D, S06.386S, S06.387A, S06.387D, S06.387S, S06.388A, S06.388D, S06.388S, S06.389A, S06.389D, S06.389S, S06.4X0A, S06.4X0D, S06.4X0S, S06.4X1A, S06.4X1D, S06.4X1S, S06.4X2A, S06.4X2D, S06.4X2S, S06.4X3A, S06.4X3D, S06.4X3S, S06.4X4A, S06.4X4D, S06.4X4S, S06.4X5A, S06.4X5D, S06.4X5S, S06.4X6A, S06.4X6D, S06.4X6S, S06.4X7A, S06.4X7D, S06.4X7S, S06.4X8A, S06.4X8D, S06.4X8S, S06.4X9A, S06.4X9D, S06.4X9S, S06.5X0A, S06.5X0D, S06.5X0S, S06.5X1A, S06.5X1D, S06.5X1S, S06.5X2A, S06.5X2D, S06.5X2S, S06.5X3A, S06.5X3D, S06.5X3S, S06.5X4A, S06.5X4D, S06.5X4S, S06.5X5A, S06.5X5D, S06.5X5S, S06.5X6A, S06.5X6D, S06.5X6S, S06.5X7A, S06.5X7D, S06.5X7S, S06.5X8A, S06.5X8D, S06.5X8S, S06.5X9A, S06.5X9D, S06.5X9S, S06.6X0A, S06.6X0D, S06.6X0S, S06.6X1A, S06.6X1D, S06.6X1S, S06.6X2A, S06.6X2D, S06.6X2S, S06.6X3A, S06.6X3D, S06.6X3S, S06.6X4A, S06.6X4D, S06.6X4S, S06.6X5A, S06.6X5D, S06.6X5S, S06.6X6A, S06.6X6D, S06.6X6S, S06.6X7A, S06.6X7D, S06.6X7S, S06.6X8A, S06.6X8D,

		S06.6X8S, S06.6X9A, S06.6X9D, S06.6X9S, S06.890A, S06.890D, S06.890S, S06.891A, S06.891D, S06.891S, S06.892A, S06.892D, S06.892S, S06.893A, S06.893D, S06.893S, S06.894A, S06.894D, S06.894S, S06.895A, S06.895D, S06.895S, S06.896A, S06.896D, S06.896S, S06.897A, S06.897D, S06.897S, S06.898A, S06.898D, S06.898S, S06.899A, S06.899D, S06.899S, S06.9X0A, S06.9X0D, S06.9X0S, S06.9X1A, S06.9X1D, S06.9X1S, S06.9X2A, S06.9X2D, S06.9X2S, S06.9X3A, S06.9X3D, S06.9X3S, S06.9X4A, S06.9X4D, S06.9X4S, S06.9X5A, S06.9X5D, S06.9X5S, S06.9X6A, S06.9X6D, S06.9X6S, S06.9X7A, S06.9X7D, S06.9X7S, S06.9X8A, S06.9X8D, S06.9X8S, S06.9X9A, S06.9X9D, S06.9X9S, S07.1XXA, S07.1XXD, S07.1XXS, Z87.820, S02.0XXA, S02.0XXB, S02.0XXD, S02.0XXG, S02.0XXK, S02.0XXS, S02.10XA, S02.10XB, S02.10XD, S02.10XG, S02.10XK, S02.10XS, S02.110A, S02.110B, S02.110D, S02.110G, S02.110K, S02.110S, S02.111A, S02.111B, S02.111D, S02.111G, S02.111K, S02.111S, S02.112A, S02.112B, S02.112D, S02.112G, S02.112K, S02.112S, S02.113A, S02.113B, S02.113D, S02.113G, S02.113K, S02.113S, S02.118A, S02.118B, S02.118D, S02.118G, S02.118K, S02.118S, S02.119A, S02.119B, S02.119D, S02.119G, S02.119K, S02.119S, S02.19XA, S02.19XB, S02.19XD, S02.19XG, S02.19XK, S02.19XS, S02.8XXA, S02.8XXB,

		S02.8XXD, S02.8XXG, S02.8XXK, S02.8XXS, S02.91XA, S02.91XB, S02.91XD, S02.91XG, S02.91XK, S02.91XS, S02.92XA, S02.92XB, S02.92XD, S02.92XG, S02.92XK, S02.92XS, S04.02XA, S04.02XD, S04.02XS, S04.031A, S04.031D, S04.031S, S04.032A, S04.032D, S04.032S, S04.039A, S04.039D, S04.039S, S04.041A, S04.041D, S04.041S, S04.042A, S04.042D, S04.042S, S04.049A, S04.049D, S04.049S, S06.0X0A, S06.0X0D, S06.0X0S, S06.0X1A, S06.0X1D, S06.0X1S, S06.0X2A, S06.0X2D, S06.0X2S, S06.0X3A, S06.0X3D, S06.0X3S, S06.0X4A, S06.0X4D, S06.0X4S, S06.0X5A, S06.0X5D, S06.0X5S, S06.0X6A, S06.0X6D, S06.0X6S, S06.0X7A, S06.0X7D, S06.0X7S, S06.0X8A, S06.0X8D, S06.0X8S, S06.0X9A, S06.0X9D, S06.0X9S, S06.1X0A, S06.1X0D, S06.1X0S, S06.1X1A, S06.1X1D, S06.1X1S, S06.1X2A, S06.1X2D, S06.1X2S, S06.1X3A, S06.1X3D, S06.1X3S, S06.1X4A, S06.1X4D, S06.1X4S, S06.1X5A, S06.1X5D, S06.1X5S, S06.1X6A, S06.1X6D, S06.1X6S, S06.1X7A, S06.1X7D, S06.1X7S, S06.1X8A, S06.1X8D, S06.1X8S, S06.1X9A, S06.1X9D, S06.1X9S, S06.2X0A, S06.2X0D, S06.2X0S, S06.2X1A, S06.2X1D, S06.2X1S, S06.2X2A, S06.2X2D, S06.2X2S, S06.2X3A, S06.2X3D, S06.2X3S, S06.2X4A, S06.2X4D, S06.2X4S,

		S06.2X5A, S06.2X5D, S06.2X5S, S06.2X6A, S06.2X6D, S06.2X6S, S06.2X7A, S06.2X7D, S06.2X7S, S06.2X8A, S06.2X8D, S06.2X8S, S06.2X9A, S06.2X9D, S06.2X9S, S06.300A, S06.300D, S06.300S, S06.301A, S06.301D, S06.301S, S06.302A, S06.302D, S06.302S, S06.303A, S06.303D, S06.303S, S06.304A, S06.304D, S06.304S, S06.305A, S06.305D, S06.305S, S06.306A, S06.306D, S06.306S, S06.307A, S06.307D, S06.307S, S06.308A, S06.308D, S06.308S, S06.309A, S06.309D, S06.309S, S06.310A, S06.310D, S06.310S, S06.311A, S06.311D, S06.311S, S06.312A, S06.312D, S06.312S, S06.313A, S06.313D, S06.313S, S06.314A, S06.314D, S06.314S, S06.315A, S06.315D, S06.315S, S06.316A, S06.316D, S06.316S, S06.317A, S06.317D, S06.317S, S06.318A, S06.318D, S06.318S, S06.319A, S06.319D, S06.319S, S06.320A, S06.320D, S06.320S, S06.321A, S06.321D, S06.321S, S06.322A, S06.322D, S06.322S, S06.323A, S06.323D, S06.323S, S06.324A, S06.324D, S06.324S, S06.325A, S06.325D, S06.325S, S06.326A, S06.326D, S06.326S, S06.327A, S06.327D, S06.327S, S06.328A, S06.328D, S06.328S, S06.329A, S06.329D, S06.329S, S06.330A, S06.330D, S06.330S, S06.331A, S06.331D, S06.331S

Tobacco use disorder	305.1	F17%
Opioid use disorder	304.0, 304.7, 305.5	F11
